# 3,3′-(*m*-Phenyl­enedi­oxy)diphthalonitrile

**DOI:** 10.1107/S1600536810011578

**Published:** 2010-04-02

**Authors:** Wei Lv, Kang Wang, Daopeng Zhang, Jianzhuang Jiang, Xiaomei Zhang

**Affiliations:** a Department of Chemistry, Shandong University, Jinan 250100, People’s Republic of China; b Department of Chemistry, University of Science and Technology Beijing, Beijing 100083, People’s Republic of China

## Abstract

In the title compound, C_22_H_10_N_4_O_2_, the dihedral angles between the mean planes of the central benzene ring and the pendant rings are 79.20 (6) and 80.29 (6)°. The dihedral angle between the pendant rings is 10.27 (7)°.

## Related literature

For background to ‘semi-rigid’ mol­ecules as ligands, see: Wang *et al.* (2005 ▶, 2009 ▶). For related structures, see: Huang *et al.* (2005 ▶); Zhang & Lu (2007 ▶). 
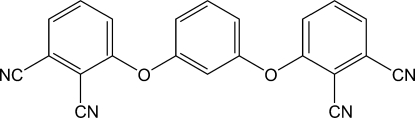


## Experimental

### 

#### Crystal data

C_22_H_10_N_4_O_2_
*M* *_r_* = 362.34
Monoclinic, 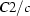

*a* = 15.668 (3) Å
*b* = 12.722 (3) Å
*c* = 19.004 (5) Å
β = 109.911 (6)°
*V* = 3561.7 (14) Å^3^
*Z* = 8
Mo *K*α radiation
μ = 0.09 mm^−1^
*T* = 298 K
0.20 × 0.15 × 0.10 mm

#### Data collection

Bruker SMART 1000 CCD diffractometer
Absorption correction: multi-scan (*SADABS*; Siemens, 1996 ▶) *T*_min_ = 0.982, *T*_max_ = 0.991
10307 measured reflections
3992 independent reflections
3145 reflections with *I* > 2σ(*I*)
*R*_int_ = 0.034

#### Refinement

*R*[*F* ^2^ > 2σ(*F* ^2^)] = 0.039
*wR*(*F* ^2^) = 0.111
*S* = 1.03
3992 reflections
254 parameters
H-atom parameters constrained
Δρ_max_ = 0.20 e Å^−3^
Δρ_min_ = −0.16 e Å^−3^

### 

Data collection: *SMART* (Siemens, 1996 ▶); cell refinement: *SAINT* (Siemens, 1996 ▶); data reduction: *SAINT*; program(s) used to solve structure: *SHELXS97* (Sheldrick, 2008 ▶); program(s) used to refine structure: *SHELXL97* (Sheldrick, 2008 ▶); molecular graphics: *SHELXTL* (Sheldrick, 2008 ▶); software used to prepare material for publication: *SHELXTL*.

## Supplementary Material

Crystal structure: contains datablocks I, global. DOI: 10.1107/S1600536810011578/hb5363sup1.cif

Structure factors: contains datablocks I. DOI: 10.1107/S1600536810011578/hb5363Isup2.hkl

Additional supplementary materials:  crystallographic information; 3D view; checkCIF report

## Figures and Tables

**Fig. 1.**
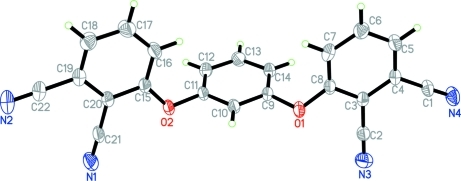
The molecular structure of (I). Displacement ellipsoids are drawn at 30% probability level.

## supplementary crystallographic information

### Comment

In the past few years, the semirigidity of molecules have been extensively employed for search of novel functional compounds. For example,a new family of multidentate O-donor ligands with a semirigid V-shaped molecular framework have been used to construct metal-organic coordination frameworks (Wang *et al.*, 2009; Wang *et al.*, 2005), in which some showed interesting properties. Here, we present the structure of a new semirigid organic ligand.

The crystal structure of the title compound is given in Fig. 1. As can be found, all the bond lengths and angles are normal and correspond to those observed in related compound (Huang *et al.*, 2005; Zhang *et al.*, 2007). The aromatic rings (C3—C8 and C15—C20) in sides of the molecule are in the same direction of the aromatic rings(C9—C14) with a cis configuration. The three dihedral angles in the title compound are 79.81Å for C3—C8 and C9—C14, 80.83Å for C15—C20 and C9—C14,and 10.54 Å for C3—C8 and C15—C20, respectively.

### Experimental

Resorcinol (0.53 g, 5 mmol) and anhydrous K_2_CO_3_ was added to the solution of 2,3-dicyanophenyl nitrate (1.73 g, 10 mmol) in DMSO (25 ml). A kind of brown solution was generated after the solution was stirred for 48 hours at room temperature. The brown solution was added to 200 ml water, and was stirred for 30 min at room temperature. The precipitate formed was filtered, and washed by water.  Yellow rods of (I) were obtained by solvent evaporation of the solution of the title compound in acetonitrile. Yield: 1.65 g, 91.2% Anal. for: C_22_H_10_N_4_O_2_ Calc. C, 72.92; H, 2.76; N, 15.47; Found: C, 72.85; H, 2.88; N, 15.44.

### Refinement

All H atoms were placed in geometrically idealized positions and treated as riding on their parent atoms with C(sp_2_ hybrid)-H distances of 0.93Å (U_iso_(H)=1.2U_eq_(C)).

### Crystal data

**Table:**

C_22_H_10_N_4_O_2_	*F*(000) = 1488
*M**_r_* = 362.34	*D*_x_ = 1.351 Mg m^−^^3^
Monoclinic, *C*2/*c*	Mo *K*α radiation, λ = 0.71073 Å
Hall symbol: -C 2yc	Cell parameters from 4716 reflections
*a* = 15.668 (3) Å	θ = 2.6–27.4°
*b* = 12.722 (3) Å	µ = 0.09 mm^−^^1^
*c* = 19.004 (5) Å	*T* = 298 K
β = 109.911 (6)°	Rod, yellow
*V* = 3561.7 (14) Å^3^	0.20 × 0.15 × 0.10 mm
*Z* = 8	

### Data collection

**Table:**

Bruker SMART 1000 CCD diffractometer	3992 independent reflections
Radiation source: fine-focus sealed tube	3145 reflections with *I* > 2σ(*I*)
graphite	*R*_int_ = 0.034
ω scans	θ_max_ = 27.6°, θ_min_ = 2.1°
Absorption correction: multi-scan (*SADABS*; Siemens, 1996)	*h* = −14→20
*T*_min_ = 0.982, *T*_max_ = 0.991	*k* = −15→15
10307 measured reflections	*l* = −24→23

### Refinement

**Table:**

Refinement on *F*^2^	Secondary atom site location: difference Fourier map
Least-squares matrix: full	Hydrogen site location: inferred from neighbouring sites
*R*[*F*^2^ > 2σ(*F*^2^)] = 0.039	H-atom parameters constrained
*wR*(*F*^2^) = 0.111	*w* = 1/[σ^2^(*F*_o_^2^) + (0.0506*P*)^2^ + 1.0114*P*] where *P* = (*F*_o_^2^ + 2*F*_c_^2^)/3
*S* = 1.03	(Δ/σ)_max_ < 0.001
3992 reflections	Δρ_max_ = 0.20 e Å^−^^3^
254 parameters	Δρ_min_ = −0.16 e Å^−^^3^
0 restraints	Extinction correction: *SHELXL97* (Sheldrick, 2008), Fc^*^=kFc[1+0.001xFc^2^λ^3^/sin(2θ)]^-1/4^
Primary atom site location: structure-invariant direct methods	Extinction coefficient: 0.0032 (4)

### Special details

**Table:**

Geometry. All esds (except the esd in the dihedral angle between two l.s. planes) are estimated using the full covariance matrix. The cell esds are taken into account individually in the estimation of esds in distances, angles and torsion angles; correlations between esds in cell parameters are only used when they are defined by crystal symmetry. An approximate (isotropic) treatment of cell esds is used for estimating esds involving l.s. planes.
Refinement. Refinement of *F*^2^ against ALL reflections. The weighted *R*-factor wR and goodness of fit *S* are based on *F*^2^, conventional *R*-factors *R* are based on *F*, with *F* set to zero for negative *F*^2^. The threshold expression of *F*^2^ > σ(*F*^2^) is used only for calculating *R*-factors(gt) etc. and is not relevant to the choice of reflections for refinement. *R*-factors based on *F*^2^ are statistically about twice as large as those based on *F*, and *R*- factors based on ALL data will be even larger.

### Fractional atomic coordinates and isotropic or equivalent isotropic displacement parameters (Å^2^)

**Table:**

	*x*	*y*	*z*	*U*_iso_*/*U*_eq_	
C2	0.24878 (8)	0.23670 (11)	0.58469 (7)	0.0496 (3)	
O1	0.41988 (6)	0.16069 (8)	0.64684 (5)	0.0597 (3)	
O2	0.73624 (5)	0.20407 (7)	0.70914 (6)	0.0582 (3)	
C9	0.49526 (7)	0.15184 (9)	0.62398 (7)	0.0425 (3)	
C10	0.57791 (7)	0.17608 (9)	0.67719 (7)	0.0437 (3)	
H10	0.5826	0.1926	0.7260	0.052*	
C20	0.88271 (8)	0.16165 (10)	0.78616 (7)	0.0438 (3)	
C11	0.65301 (7)	0.17492 (9)	0.65542 (7)	0.0459 (3)	
C14	0.48704 (8)	0.12738 (10)	0.55183 (7)	0.0474 (3)	
H14	0.4308	0.1110	0.5168	0.057*	
C3	0.26451 (7)	0.12567 (10)	0.58820 (6)	0.0420 (3)	
C4	0.19286 (7)	0.05388 (10)	0.56020 (6)	0.0443 (3)	
C15	0.79734 (7)	0.12689 (9)	0.74065 (7)	0.0448 (3)	
C12	0.64815 (9)	0.15132 (11)	0.58405 (8)	0.0554 (3)	
H12	0.7000	0.1512	0.5707	0.067*	
N3	0.23572 (10)	0.32515 (11)	0.58276 (8)	0.0727 (4)	
C21	0.90124 (8)	0.27171 (11)	0.79679 (8)	0.0535 (3)	
C8	0.35196 (8)	0.08751 (11)	0.61986 (7)	0.0466 (3)	
C16	0.77924 (9)	0.02090 (10)	0.73059 (8)	0.0558 (3)	
H16	0.7222	−0.0022	0.7005	0.067*	
C5	0.20892 (9)	−0.05263 (11)	0.56674 (8)	0.0528 (3)	
H5	0.1612	−0.1000	0.5485	0.063*	
C19	0.95031 (8)	0.08725 (11)	0.82021 (7)	0.0499 (3)	
C13	0.56427 (9)	0.12762 (11)	0.53235 (8)	0.0543 (3)	
H13	0.5597	0.1115	0.4835	0.065*	
N2	1.11044 (8)	0.15377 (13)	0.89675 (8)	0.0774 (4)	
C1	0.10262 (8)	0.09249 (11)	0.52163 (8)	0.0524 (3)	
C6	0.29672 (9)	−0.08846 (11)	0.60074 (8)	0.0585 (3)	
H6	0.3077	−0.1604	0.6061	0.070*	
C18	0.93170 (10)	−0.01859 (12)	0.80996 (8)	0.0630 (4)	
H18	0.9762	−0.0681	0.8328	0.076*	
C17	0.84594 (11)	−0.05017 (12)	0.76525 (9)	0.0661 (4)	
H17	0.8332	−0.1216	0.7585	0.079*	
C22	1.03960 (9)	0.12385 (12)	0.86413 (8)	0.0577 (4)	
N4	0.03136 (8)	0.12171 (11)	0.48961 (8)	0.0725 (4)	
C7	0.36803 (9)	−0.01905 (12)	0.62679 (8)	0.0564 (3)	
H7	0.4269	−0.0441	0.6490	0.068*	
N1	0.91780 (9)	0.35902 (11)	0.80612 (9)	0.0809 (4)	

### Atomic displacement parameters (Å^2^)

**Table:**

	*U*^11^	*U*^22^	*U*^33^	*U*^12^	*U*^13^	*U*^23^
C2	0.0399 (6)	0.0575 (8)	0.0501 (7)	−0.0042 (6)	0.0137 (5)	−0.0057 (6)
O1	0.0322 (4)	0.0797 (7)	0.0681 (6)	−0.0119 (4)	0.0181 (4)	−0.0269 (5)
O2	0.0293 (4)	0.0448 (5)	0.0869 (7)	−0.0052 (3)	0.0020 (4)	−0.0088 (4)
C9	0.0287 (5)	0.0456 (6)	0.0512 (7)	−0.0002 (4)	0.0111 (5)	−0.0022 (5)
C10	0.0347 (6)	0.0455 (6)	0.0463 (6)	−0.0043 (5)	0.0080 (5)	−0.0018 (5)
C20	0.0333 (6)	0.0515 (7)	0.0468 (6)	−0.0069 (5)	0.0137 (5)	−0.0004 (5)
C11	0.0286 (5)	0.0389 (6)	0.0635 (8)	−0.0048 (4)	0.0071 (5)	−0.0023 (5)
C14	0.0357 (6)	0.0514 (7)	0.0482 (7)	−0.0012 (5)	0.0056 (5)	−0.0031 (5)
C3	0.0334 (5)	0.0526 (7)	0.0406 (6)	−0.0027 (5)	0.0134 (5)	−0.0007 (5)
C4	0.0323 (6)	0.0554 (7)	0.0442 (6)	−0.0035 (5)	0.0117 (5)	0.0026 (5)
C15	0.0326 (5)	0.0463 (7)	0.0540 (7)	−0.0043 (5)	0.0129 (5)	−0.0004 (5)
C12	0.0422 (7)	0.0545 (7)	0.0764 (9)	−0.0084 (6)	0.0290 (6)	−0.0085 (7)
N3	0.0745 (9)	0.0599 (8)	0.0802 (9)	0.0003 (7)	0.0219 (7)	−0.0093 (6)
C21	0.0306 (6)	0.0589 (8)	0.0642 (8)	−0.0086 (5)	0.0076 (5)	−0.0030 (6)
C8	0.0313 (5)	0.0621 (8)	0.0462 (6)	−0.0049 (5)	0.0128 (5)	−0.0072 (5)
C16	0.0443 (7)	0.0484 (7)	0.0661 (8)	−0.0098 (6)	0.0077 (6)	0.0010 (6)
C5	0.0432 (7)	0.0544 (8)	0.0602 (8)	−0.0089 (6)	0.0168 (6)	0.0020 (6)
C19	0.0383 (6)	0.0640 (8)	0.0453 (6)	−0.0011 (6)	0.0116 (5)	0.0054 (6)
C13	0.0537 (7)	0.0589 (8)	0.0534 (7)	−0.0065 (6)	0.0223 (6)	−0.0070 (6)
N2	0.0416 (7)	0.1071 (11)	0.0731 (8)	−0.0068 (7)	0.0059 (6)	0.0123 (8)
C1	0.0369 (6)	0.0569 (8)	0.0581 (7)	−0.0078 (6)	0.0094 (6)	0.0031 (6)
C6	0.0523 (8)	0.0527 (8)	0.0718 (9)	0.0046 (6)	0.0229 (7)	0.0087 (7)
C18	0.0578 (8)	0.0600 (9)	0.0630 (8)	0.0099 (7)	0.0098 (7)	0.0136 (7)
C17	0.0674 (9)	0.0459 (7)	0.0737 (9)	−0.0043 (6)	0.0093 (8)	0.0075 (7)
C22	0.0395 (7)	0.0768 (10)	0.0532 (7)	0.0029 (6)	0.0111 (6)	0.0111 (7)
N4	0.0399 (6)	0.0705 (8)	0.0914 (10)	−0.0021 (6)	0.0018 (6)	0.0083 (7)
C7	0.0360 (6)	0.0694 (9)	0.0617 (8)	0.0092 (6)	0.0140 (6)	0.0056 (7)
N1	0.0538 (7)	0.0594 (8)	0.1145 (12)	−0.0151 (6)	0.0094 (8)	−0.0088 (8)

### Geometric parameters (Å, °)

**Table:**

C2—N3	1.1421 (19)	C15—C16	1.3777 (18)
C2—C3	1.4315 (19)	C12—C13	1.3798 (19)
O1—C8	1.3750 (15)	C12—H12	0.9300
O1—C9	1.3949 (14)	C21—N1	1.1406 (19)
O2—C15	1.3596 (15)	C8—C7	1.377 (2)
O2—C11	1.4048 (14)	C16—C17	1.369 (2)
C9—C14	1.3686 (17)	C16—H16	0.9300
C9—C10	1.3795 (16)	C5—C6	1.3828 (19)
C10—C11	1.3740 (16)	C5—H5	0.9300
C10—H10	0.9300	C19—C18	1.377 (2)
C20—C15	1.3946 (16)	C19—C22	1.4406 (18)
C20—C19	1.4035 (18)	C13—H13	0.9300
C20—C21	1.4303 (19)	N2—C22	1.1369 (18)
C11—C12	1.3657 (19)	C1—N4	1.1368 (16)
C14—C13	1.3808 (18)	C6—C7	1.377 (2)
C14—H14	0.9300	C6—H6	0.9300
C3—C8	1.3834 (16)	C18—C17	1.382 (2)
C3—C4	1.4038 (16)	C18—H18	0.9300
C4—C5	1.3761 (19)	C17—H17	0.9300
C4—C1	1.4393 (17)	C7—H7	0.9300
			
N3—C2—C3	178.96 (15)	N1—C21—C20	178.61 (16)
C8—O1—C9	117.33 (9)	O1—C8—C7	122.54 (11)
C15—O2—C11	118.00 (9)	O1—C8—C3	116.79 (12)
C14—C9—C10	121.99 (11)	C7—C8—C3	120.59 (11)
C14—C9—O1	121.98 (10)	C17—C16—C15	119.49 (12)
C10—C9—O1	115.89 (11)	C17—C16—H16	120.3
C11—C10—C9	117.60 (11)	C15—C16—H16	120.3
C11—C10—H10	121.2	C4—C5—C6	119.28 (12)
C9—C10—H10	121.2	C4—C5—H5	120.4
C15—C20—C19	119.07 (12)	C6—C5—H5	120.4
C15—C20—C21	120.25 (11)	C18—C19—C20	120.26 (12)
C19—C20—C21	120.67 (11)	C18—C19—C22	121.00 (13)
C12—C11—C10	122.52 (11)	C20—C19—C22	118.73 (13)
C12—C11—O2	120.25 (11)	C12—C13—C14	121.25 (12)
C10—C11—O2	117.16 (11)	C12—C13—H13	119.4
C9—C14—C13	118.43 (11)	C14—C13—H13	119.4
C9—C14—H14	120.8	N4—C1—C4	178.29 (16)
C13—C14—H14	120.8	C7—C6—C5	120.85 (13)
C8—C3—C4	118.83 (12)	C7—C6—H6	119.6
C8—C3—C2	119.73 (11)	C5—C6—H6	119.6
C4—C3—C2	121.43 (10)	C19—C18—C17	119.06 (13)
C5—C4—C3	120.56 (11)	C19—C18—H18	120.5
C5—C4—C1	119.98 (11)	C17—C18—H18	120.5
C3—C4—C1	119.41 (12)	C16—C17—C18	121.77 (14)
O2—C15—C16	124.39 (11)	C16—C17—H17	119.1
O2—C15—C20	115.27 (10)	C18—C17—H17	119.1
C16—C15—C20	120.34 (11)	N2—C22—C19	177.82 (15)
C11—C12—C13	118.21 (12)	C8—C7—C6	119.83 (12)
C11—C12—H12	120.9	C8—C7—H7	120.1
C13—C12—H12	120.9	C6—C7—H7	120.1
			
C8—O1—C9—C14	41.14 (17)	C9—O1—C8—C3	−129.48 (12)
C8—O1—C9—C10	−143.02 (12)	C4—C3—C8—O1	−179.70 (10)
C14—C9—C10—C11	−0.04 (18)	C2—C3—C8—O1	0.21 (16)
O1—C9—C10—C11	−175.87 (11)	C4—C3—C8—C7	−2.83 (18)
C9—C10—C11—C12	0.24 (18)	C2—C3—C8—C7	177.08 (12)
C9—C10—C11—O2	177.36 (10)	O2—C15—C16—C17	−179.83 (13)
C15—O2—C11—C12	−77.49 (16)	C20—C15—C16—C17	−0.4 (2)
C15—O2—C11—C10	105.33 (13)	C3—C4—C5—C6	−0.51 (19)
C10—C9—C14—C13	−0.24 (19)	C1—C4—C5—C6	176.87 (12)
O1—C9—C14—C13	175.35 (12)	C15—C20—C19—C18	−1.39 (19)
C8—C3—C4—C5	2.48 (17)	C21—C20—C19—C18	179.66 (13)
C2—C3—C4—C5	−177.43 (11)	C15—C20—C19—C22	176.97 (12)
C8—C3—C4—C1	−174.92 (11)	C21—C20—C19—C22	−1.98 (18)
C2—C3—C4—C1	5.17 (17)	C11—C12—C13—C14	−0.1 (2)
C11—O2—C15—C16	−8.46 (19)	C9—C14—C13—C12	0.3 (2)
C11—O2—C15—C20	172.12 (11)	C4—C5—C6—C7	−1.1 (2)
C19—C20—C15—O2	−179.22 (11)	C20—C19—C18—C17	0.6 (2)
C21—C20—C15—O2	−0.27 (17)	C22—C19—C18—C17	−177.77 (14)
C19—C20—C15—C16	1.32 (19)	C15—C16—C17—C18	−0.4 (2)
C21—C20—C15—C16	−179.72 (13)	C19—C18—C17—C16	0.4 (2)
C10—C11—C12—C13	−0.2 (2)	O1—C8—C7—C6	177.91 (12)
O2—C11—C12—C13	−177.18 (11)	C3—C8—C7—C6	1.2 (2)
C9—O1—C8—C7	53.72 (17)	C5—C6—C7—C8	0.8 (2)
